# Brain networks for visual creativity: a functional connectivity study of planning a visual artwork

**DOI:** 10.1038/srep39185

**Published:** 2016-12-19

**Authors:** Nicola De Pisapia, Francesca Bacci, Danielle Parrott, David Melcher

**Affiliations:** 1 CIMeC - Center for Mind/Brain Sciences, University of Trento, Italy; 2 Department of Psychology and Cognitive Science, University of Trento, Rovereto (TN), Italy; 3 Museo d’Arte Moderna e Contemporanea di Trento e Rovereto (Mart), Rovereto, Italy; 4 Department of Art, University of Tampa, Tampa, Florida, USA

## Abstract

Throughout recorded history, and across cultures, humans have made visual art. In recent years, the neural bases of creativity, including artistic creativity, have become a topic of interest. In this study we investigated the neural bases of the visual creative process with both professional artists and a group of control participants. We tested the idea that creativity (planning an artwork) would influence the functional connectivity between regions involved in the default mode network (DMN), implicated in divergent thinking and generating novel ideas, and the executive control network (EN), implicated in evaluating and selecting ideas. We measured functional connectivity with functional Magnetic Resonance Imaging (fMRI) during three different conditions: rest, visual imagery of the alphabet and planning an artwork to be executed immediately after the scanning session. Consistent with our hypothesis, we found stronger connectivity between areas of the DMN and EN during the creative task, and this difference was enhanced in professional artists. These findings suggest that creativity involves an expert balance of two brain networks typically viewed as being in opposition.

Making visual artwork, such as drawings and sculptures, is an activity that has accompanied human cognition across cultures since the earliest records of human activity. Tens of thousands of years ago, human ancestors created artifacts that depicted real and imagined creatures, such as animals or deities, and developed complex representations with more abstract meanings such as collections of lines and shapes. The widespread nature of art, across cultures and history, is a fascinating aspect of human cognition. For example, one key aspect of art is that it must be created in the mind of the artist so that these intentions can be translated into a physical form. This involves both a planning process, during which the artist decides what she or he will do, and the execution of the artwork, during which time artists typically revise and update these plans^1^,^2^,^3^,^4^,^5^. The source of this artistic ability has had many explanations throughout history, ranging from external/divine inspiration from gods or the Muses to the route imitation of nature, to artistic “genius”^6^. Current thinking on art making in Western cultures, since the Renaissance and the Enlightenment, tends to emphasize imagination and the human capacity for creative thinking^6^,^7^,^8^,^9^.

Creativity is roughly definable as the process of generating novel and worthwhile ideas or objects and is thought to involve several types of cognitive abilities. One widespread idea is that creativity must involve both novelty (new ideas or objects are the outcome) and usefulness (the new idea/object must be worthwhile)^6^,^8^. There is widespread consensus that creativity is a fundamental and valuable part of human cognition and the scientific study of creativity has exploded in the last few decades. However, neurocognitive research on creativity is still at a very early stage, and no clear consensus has been reached on its definition, or on a cognitive model of how individuals generate new ideas, solutions or works of art.

## Creativity, cognitive control and spontaneous thinking

One recent line of research investigates the possibility that at least some forms of creativity depend on the joint engagement of two types of cognitive processes that might be seen as otherwise working in competition: executive control and spontaneous thinking^1^. Executive control describes the processes that regulate mental resources. In contrast, spontaneous thinking is instead a mode of mental activity in which the association between the flow of thoughts, emotions, images, sounds and so on is less regulated, in a continuous stream of internal processing. This mode of thinking appears necessary for the production of new ideas by exploring new associations and combinations of possibilities. By putting these two processes together, executive control allows for the selection and evaluation of ideas that are generated via spontaneous thinking to check if the new combinations can be executed or not, and at what costs, and whether they successfully solve a problem or express what was intended.

In this study we explored the relationship between these two mental processes–control of attention and mind-wandering– and their neuronal bases during a creative task. Specifically, we investigated the planning and generation of a work of visual art on an assigned topic. Thus, the first aim of our study was to characterize brain activity during the creative process of generating the idea for a new artwork. Additionally, we investigated whether the brain mechanisms involved during the execution of this task depended on the artistic skills and experience of the individuals. Hence, half of our participants were professional artists, with extensive education and professional activity in the field of visual arts, while the other participants were not trained or practicing artists.

## Brain networks for creativity

As mentioned above, creativity is currently viewed as a highly complex function that requires several skills. It is thought to require both novelty, the process of generating something new, and usefulness, which indicates an evaluative process. This suggests that, rather than there being a single “creativity module” in the brain, distributed networks of brain regions may be necessary to generate original and useful ideas^10^. More generally, although cognitive neuroscience research has often assumed that thinking could be explained by looking at activity in isolated brain regions, more recently the perspective has shifted towards the view that cognitive functions should be studied as resulting from large-scale network interactions^11^.

In the current study, the two networks of interest are the executive control (EN) and default-mode-(DMN) networks. ENs are involved when individuals are engaged in reaching a goal, when there is a need to follow rules and evaluate outcomes, and when resources have to converge in the attempt to find a valid solution to a problem. The key brain hub of this network is thought to include lateral nodes of the dorsolateral prefrontal cortex (anterior and posterior), which display the capacity to enable the formation, control and selection of mental representations according to internal goals^12^. These prefrontal properties have been implicated in creativity as well as other abilities such as cognitive control, abstract thinking, decision making and planning^13^,^14^,^15^,^16^,^17^ Other nodes of the ENs include the anterior cingulate cortex and portions of the attentional control system, such as the superior parietal lobes.

In contrast, the brain network that tends to be active during spontaneous processing, independent of current external stimuli, is the DMN^18^. This network has been found to be linked to activities such as spontaneous thinking, mind-wandering or divergent thinking. The DMN includes the medial prefrontal cortex (anterior part of the medial frontal gyrus), the posterior cingulate cortex, the medial temporal lobes, the precuneus and the temporo-parietal junction.

When humans perform stimulus-based tasks, it has been consistently found that activity increases in the ENs and decreases in the DMNs^19^,^20^,^21^. Thus, these two networks are often viewed as working in opposition, or in a sort of “push-pull” relationship^22^. However, tasks that involve creativity may involve both “divergent” thinking (spontaneous and free-ranging) and “convergent” thinking (control and evaluation) at the same time^23^. Thus, one hypothesis is that creativity involves these two networks working together^24^. Support for this idea comes from studies using different tasks which include a visual creative task^1^,^22^, mind wandering^25^, narrative speech comprehension^26^, autobiographical planning^27^, insight problem solving^28^,^29^, and a “fluid” analogy task^30^. One recent study reported that a reduction in task-induced deactivation in the precuneus was associated with higher creativity as measured by divergent thinking^31^. This finding suggests that creativity might involve a reduction in the competition between EN and DMN activity.

## Expertise, neuroplasticity and artists

Historically, artistic abilities were often assigned to external factors (divine inspiration) or to artistic “genius”^32^. Currently, however, it is widely believed that artistic ability, and creativity more generally, exists to varying degrees in most, if not all, people. At the same time, some people have greater abilities and expertise for certain creative tasks. For example, leading scientists and artists – each in their respective field - are considered as individuals who have special skills and distinct abilities for creating ideas or objects. These capacities are generally viewed as a combination of personal talent and many years of training^33^,^34^. In the field of cognitive neuroscience, the brain is viewed as a plastic organ which constantly adapts to the environment and to the activity it performs^35^. Developing expertise in, for example, playing a musical instrument^36^, driving a taxi^37^ or memorizing large texts via verbal recitation^38^ changes brain structures and functionalities in the specific brain areas that are used in these tasks. Similarly we can expect that professionals in the visual arts have developed a higher efficiency in the brain areas involved in planning and making artworks. We investigate the idea that a high level of artistic ability requires an expert use of the simultaneous engagement of two basic modes of brain functioning, namely mind-wandering and the control of attention, that involve two very different brain networks that are often viewed as being in opposition.

## The current experiment

The current study was motivated by the goal of studying the creative phase in which the *production* of ideas (emphasizing novelty and free thinking) and selection of these ideas (requiring control and evaluation) interact. We compared neural activity in three tasks: (1) a control block involving mind-wandering and defocused thinking, (2) another control block involving a simple task and focused thinking, and (3) a key experimental block involving creative visualization, idea generation and planning. Our design followed the approach of previous studies, in particular those looking at musical creativity and improvisation^4^,^5^,^39^, by including one condition that required repeating a learned pattern as well as another condition required generating a new and original artistic product. The logic of these studies is that the difference between repeating a learned output and creating a new output is likely to involve the creative process^4^,^5^,^39^. We then looked at how brain regions of artists and non-artists worked in the various blocks, and how these regions were functionally connected. In other words, we characterized interregional neural interactions and how key brain regions previously identified in the DMN and in EN worked together in concert.

To this end, we recruited a group of twelve professional artists, whose main techniques include drawing (artists group). Additionally, we recruited a control group of twelve volunteers matched for gender, age and education to the artist group, but whose profession and skills were not linked to drawing (control group). For the control tasks, participants were asked in a first block to think about whatever they liked, without focusing on anything in particular; in the second control task they had to mentally visualize the letters of the alphabet, from A to Z, and imagine to draw them, in no special font, just plain letters. This was a visual version of the task used in previous studies in which participants repeat a learned pattern^4^,^5^,^39^. In the experimental block, the participants were instructed that they had eight minutes to mentally create and visualize a drawing on the topic of “landscape”. They were informed that they had then to actually draw what they thought afterwards in a special room that was prepared for this purpose, with pencils, pens, special papers and other artistic material. This condition was a variant on creative tasks that focused specifically on creativity in visual art. We specified that “landscape” could be interpreted in many ways, namely as a geographical landscape, but also metaphorical, thus with the general understanding that they had freedom to visualize and create whatever they preferred: it was an open-ended task with weak constraints other than needing to plan the visual artwork.

The design of this experiment was motivated by the goal of studying a visually creative phase in which idea production and selection interact, compared with a resting state condition and a non- creative non-resting state condition. We investigated how brain regions of artists and non-artists worked, comparatively, across the separate blocks, and how functionally connected brain regions between the two groups differed, with a main goal of characterizing interregional neural interactions and how they worked in synchrony during the different tasks. The analyses that we report here concern the coordination between brain networks during the various experimental conditions (i.e., rest, alphabet visualization and the creative task). This type of analysis looks at correlations of activities in the whole brain of every participant during the various phases. This method takes the name of “functional connectivity”, and it relies on the hypothesis that there is coordination of activity between different brain regions in order to achieve a task^40^.

**Figure 1:**
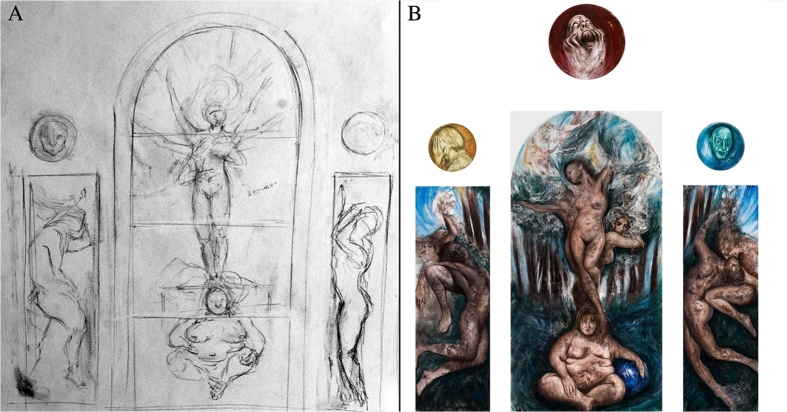
Example of drawings and paintings Example of the initial sketch. (**A**) and final artwork (**B**) on display at the MART museum, of one of the 12 professional artists who participated in the study (Jacopo Dimastrogiovanni, work entitled “Simulacra – Human Landscape”). The sketch and final work were both included in the exhibition entitled “In resonance: snapshots of creativity in the brain” (In risonanza: istantanee di creatività nel cervello) at the Museum of Contemporary and Modern Art of Rovereto and Trento (MART) Italy. Images reproduced with permission of the artist.

Note that all of the artists completed the drawing process that they planned in the MRI scanner immediately after the scanning session. The artists brought their own tools, but they were also provided with materials such as pens and paper. An example of these drawings, completed at the neuroimaging center in a nearby room, is shown in Fig. 1A. In addition, each of the artists completed their planned artwork over a subsequent period of weeks after the experiment took place in their own studio space and a number of these works were then selected for a museum exhibition entitled “In resonance: snapshots of creativity in the brain” (*In risonanza: istantanee di creatività nel cervello*) at the Museum of Contemporary and Modern Art of Rovereto and Trento (MART) in Italy. As shown in Fig. 1B, the more complete versions of the artwork often incorporated other materials and methods that went beyond the initial drawing.

## Materials and Methods

### Participants

24 participants (14 male, 10 female) were matched for age and education between the experimental and control groups. Participants were all right handed and had no history of CNS-affecting drugs or neurological disease. The experimental group was composed of 12 professional artists whose main techniques included drawing; the control group was composed of 12 non-artists, whose profession and skills were not related to drawing. Professional artists were recruited through the Museum of Modern Art (MART) in Rovereto (Italy), whereas controls (non-professional artists) were recruited through local advertisements. All sections of the experiment were performed in accordance with relevant guidelines and regulations. This study was approved by The University of Trento Ethics Committee on Experiments Involving Human Beings. Informed consent was obtained from all participants. They also received a reimbursement for their travel expenses to reach the neuroimaging laboratory.

### Behavioral Assessment and Scanning Procedure

The subjects were screened using the Vividness of Visual Imagery Questionnaire^41^, a self-assessment of visual imagery ability. Participants were asked to rate their experience of visual imagery on 16 questions, ranging on a scale from 1 to 5, where 1 signified “Perfectly clear and as vivid as normal vision” and a 5, “No image at all (only “knowing” that you are thinking of the object)”.

Participants were pre-screened for MRI compatibility prior to entering the magnetic environment by a medical doctor, complied with all safety precautions, and gave consent to participate in the experiment. They were given brief instructions about what to expect during the session before entering in the scanner, and were informed during the session regarding task instructions through an intercom device.

The scan consisted of both anatomical and functional imaging data. Anatomical data was collected for the first 6 minutes of the session, followed by functional imaging in the experimental session. The experimental session consisted of three blocks, each approximately 8 minutes in duration, all performed with closed eyes: Resting State (control block 1), Alphabet reproduction Task (control block 2), and the Creative Task (experimental block). During the Resting State block participants were instructed to rest, but not sleep, allowing their mind to wander without focusing on anything in particular. The second block consisted of the Alphabet task in which participants were told to mentally visualize the letters of the alphabet from A to Z (and then back to A if there was still time) in a plain font (no embellishments), keeping a slow and regular pace for the 8 minute duration. Thus, participants merely repeated a learned sequence in order to produce a visual image without the need to create novel contents. The exact details of the third block, the Creative Task, were only revealed to the participants while in the scanner so that they could not plan or generate ideas prior to the scanning session. Specific instructions on the topic for the creative block were given therefore just prior to the functional scanning, and they were as follows. Participants were told to mentally create and visualize a new drawing with the topic of “landscape” in the subsequent 8 minute scanning session, and that they would need to recreate this novel artistic visualization once they left the scanner, in a specially designated room. The topic “landscape” was left open-ended, such that this could refer to a geographic landscape or a metaphoric landscape, allowing for a broader range of artistic expression.

The two control tasks allowed us to contrast brain activity during the creative task while controlling for purely mind-wandering activities not directed towards a goal (Resting State) and for mental visualization of a learned pattern during a goal-directed task without a creative component (Alphabet Task). This contrast between visualizing a learned pattern (the Alphabet Task) and generating a completely novel visual pattern (Creative Task) followed the logic of previous studies comparing repeating a musical or spoken sequence with creatively improvising a novel musical or spoken composition^4^,^5^,^39^.

Participants were given artistic materials to create their visualized works after the completion of the scan, after completing a brief questionnaire on their scanning experience. This questionnaire related to their experience of both comfort and task related activities in the MRI during the resting, alphabet, and creative phases. Most subjects reported in the questionnaire that they found the environment conducive for all phases of the experiment and that they were relatively comfortable in the scanning environment. All participants indicated compliance with instructions and the ability to carry out the three required tasks.

### Anatomical and Functional Imaging Acquisition

Imaging data were acquired using a 4 T Bruker MedSpec Biospin MR scanner and a birdcage transmit, 8-channel radio-frequency head receiver coil. Head motion was restricted using foam padding surrounding the head. Functional images were acquired with a single shot T2*-weighted gradient-recalled echo-planar imaging (EPI) sequence. 37 slices were acquired in ascending interleaved order, slightly tilted to run parallel to the calcarine sulcus, with a TR (time to repeat) of 2200 ms (voxel resolution, 3 × 3 × 3 mm3; TE (time to echo), 33 ms; flip angle (FA), 75°; field of view (FOV), 192 × 192 mm2; slice gap, 0.4 mm). Each run consisted of 200 volumes. To correct for distortions in geometry and intensity in the EPI images, we applied distortion correction on the basis of the PSF (point-spread function) data acquired before the EPI scans^42^. There were five dummy scans. To be able to co-register the low-resolution functional images to a high-resolution anatomical scan, we acquired a T1 weighted anatomical scan (MP-RAGE; 1 × 1 × 1 mm3; FOV, 256 × 224 mm2; 176 slices; GRAPPA acquisition with an acceleration factor of 2; TR, 2700 ms; TE, 4.18 ms; inversion time (TI), 1020 ms; 7° flip angle). Imaging data were then slice-time corrected and realigned using the Statistical Parametric Mapping (SPM) 8 package (Wellcome Institute of Cognitive Neurology, London). Functional volumes were co-registered and resliced to a voxel size of 2 mm^3^, normalized to the MNI template brain (Montreal Neurological Institute), and smoothed with an 8 mm^3^ isotropic Gaussian kernel.

### Connectivity Analysis

Connectivity analysis was performed using the CONN toolbox (http://www.nitrc.org/projects/conn^43^). CONN implements the CompCor method^44^ to identify principal components associated with segmented white matter (WM) and cerebrospinal fluid (CSF). WM, CSF, and realignment parameters were entered as confounds in a first-level analysis, and the data were band-pass filtered to 0.008 Hz–0.09 Hz. CompCor addresses the confounding effects of subject movement without affecting intrinsic functional connectivity^45^, thus global signal was not regressed.

**Figure 2:**
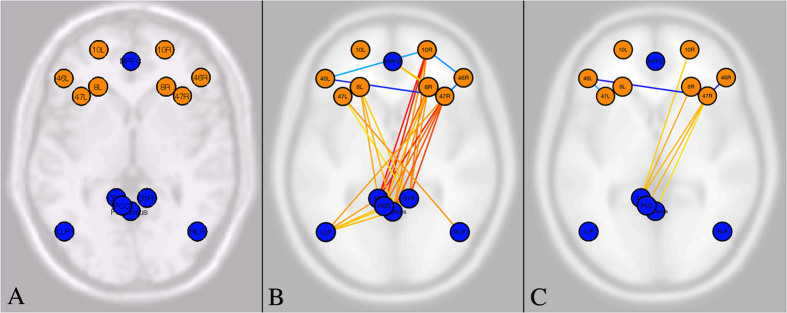
Functional connectivity during creativity. (**A**) Regions of interest (ROI) for second level and seed-to-voxel analyses. The nodes selected for the executive control network (orange nodes) were: (a) bilateral anterior dorsolateral prefrontal cortex (10L and 10R), (b) bilateral dorsolateral prefrontal cortex (46L and 46R), (c) bilateral frontal eye fields (8L and 8R), (d) bilateral Inferior frontal Gyrus (47L and 47R). For the default-mode network (blue nodes) we selected: (a) medial prefrontal cortex, (b) three nodes of posterior cingulate cortex (31L and 31R), (c) precuneus, (d) bilateral temporo-parietal junction (LLP and RLP). In the labels for the regions locations in figure, the numbers refer to the corresponding Brodmann areas (e.g., 10 is BA 10), L refers to left hemisphere, and R refers to right hemisphere. (**B**) Functional connectivity (all participants) between EN (orange) and DMN (blue) nodes in the Creative Task versus Resting State (FDR corr, p > 0.05). Red/orange connections denote increased connectivity in the Creative Task versus Resting State; Blue/cyan connections denote decreased connectivity in the Creative Task versus Resting State. For exact t-values and p-values, see Table 2. (**C**) Functional connectivity (all participants) between EN (orange) and DMN (blue) nodes in the Creative Task versus Alphabet Task (FDR corr, p > 0.05). Red/orange connections denote increased connectivity in the Creative Task versus the Alphabet Task; Blue/cyan connections denote decreased connectivity in the Creative Task versus the Alphabet task. For exact t-values and p-values, see Table 3.

After this preliminary processing, we conducted a series of second-level region of interest (ROI) analyses, and seed-to-voxel analyses. We used the ROI analysis to test the functional connectivity between the main prefrontal nodes of the EN and the main nodes of the DMN. In particular (see Fig. 2A), the nodes selected were for the EN (a) anterior dorsolateral prefrontal cortex (bilateral BA 10), (b) dorsolateral prefrontal cortex (bilateral BA 46), (c) frontal eye fields (bilateral BA 8), (d) inferior frontal gyrus (bilateral BA 47); for the DMN we selected (a) medial prefrontal cortex, (b) three nodes of posterior cingulate cortex (bilateral BA 31), (c) precuneus, (d) temporo-parietal junction.

To define these regions we used seven 10 mm spherical clusters with peak-coordinates based on structural data^43^ and analysis of resting-state data^45^. See Table 1 for approximate centers of node coordinates in MNI (Montreal Neurological Institute) space and conditions tested. Temporal correlations between BOLD signals in the seed ROIs were computed by CONN. These correlations were compared between conditions (Creative Task vs. Resting State; Creative Task vs. Alphabet Task) for all participants, and between subject populations by single conditions (Artist vs. Non-artist during Resting state, Alphabet Task, and Creative Task individually). Specifically, t-tests and Fisher’s Z-transformed correlations were computed to analyze differences in functional connectivity between the seed and target ROIs across groups. ROI-to-ROI results are reported when significant at a level of p < 0.05, false discovery rate (FDR) corrected^46^. This analysis determines the amount of correlation between regions, and it computes the functional connectivity between pairs of ROIs, and thus it does not give any directional information. To explore the directionality of the connectivity of the key hubs (the most functionally connected regions), we also computed a multivariate regression analyses on ROI pairs for the most connected regions. This approach allows for an estimation of the relative strength of information flow between any two units. The method informs on which ROI pushes the relative signal change within a pair of regions (effective connectivity analysis)^47^. Specifically, we examined whether a source region predicts more likely the percent signal change in a target region (but not viceversa). The effect size represents the percentage change in BOLD activity in the target region associated with the percentage change of BOLD activity in the source region. It is possible therefore to compute a “difference score” between pairs of ROIs, which results in a directionality measure representing which ROI is likely to drive the activity in a pair of ROIs^48^. The ROI-to-ROI results of effective connectivity were reported when significant at a level of p < 0.05, false discovery rate (FDR) corrected.

Seed-to-voxel analyses were then computed in an effort to explore which regions outside of the DMN and attention network nodes were correlated with creative and non-creative tasks, differentially specifically in the artists versus the non-artists. Seed regions used were the main nodes of the DMN and the EN resulting in the ROI-to-ROI analyses, computed for temporal correlations for activity in other voxels in the brain. Significance level was set at a voxel-wise threshold of p < 0.001 uncorrected and cluster-level threshold at p < 0.05 FDR corrected.

## Results

### Imagery Vividness Questionnaire

The difference between artists and non-artists in vividness of their imagery (lower scores indicate higher imagery vividness: Control Mean = 36.5, STD = 6.85; Artists Mean = 29.19, STD = 7.07) was significant (t-test; p-value = 0.0199). This result confirms our expectation that artists tend to use visual imagery more vividly than non-artists.

### Functional neuroimaging

#### Creativity vs Rest (all participants)

In the first ROI-to-ROI analysis we examined the functional connectivity between EN and DMN nodes in the Creative Task versus Resting State (see Table 2 and Fig. 2B). This revealed significantly stronger connectivity during creativity between nodes of the two networks, decreased connectivity within the EN (in particular notice between bilateral BA46 and right BA47), and increased connectivity within the DMN. This suggests that the ENs and DMNs are more strongly connected during the visually creative stages compared to pure resting mental activities, and the EN is less functionally connected with activity of the right BA47, a prefrontal node considered to be involved in inhibitory control^49^.

Right BA47 appeared to be a hub node in this comparison, with the highest number of significant functional connections with other nodes (8 connections). With effective connectivity analysis we found that this region was significantly effective in predicting changes in activity in DMN nodes, with effective connectivity towards left BA 31 (beta = 0.23, p < 0.0004 corr), right BA31 (beta = 0.29, p = 0.0004 corr), precuneus (beta = 0.51, p = 0.001 corr), posterior cingulate cortex (beta = 0.34, p = 0.004 corr), left lateral parietal (beta = 0.45, p = 0.007 corr), medial prefrontal cortex (beta = 0.28, p = 0.02 corr). Vice-versa, DMN nodes did not have an predictive effect on right BA 47, except posterior cingulate cortex with a weaker effect (beta = 0.12, p = 0.01 corr) compared to the size of the effect coming from the right BA47.

#### Creativity vs Alphabet (all participants)

In the second ROI-to-ROI analysis we examined the functional connectivity between EN nodes and DMN nodes in the Creative Task versus the Alphabet task (see Table 3 and Fig. 2C). This analysis revealed significantly stronger connectivity during creativity between the EN and DMN, and decreased connectivity within the EN (again between bilateral BA46 and right BA47), as in the previous contrast Creative Task versus Resting State. This suggests confirms that ENs and DMNs are more strongly connected during the visually creative phase also when comparing with an alphabet visualization task - a mental activity which is close to visualization efforts of the Creative Task, but without the creative component. This finding is consistent with previous investigations of musical creativity comparing piano and rap improvisation to repeating learned sequences^4^,^5^,^39^.

As in the Creativity versus Rest comparison, also in this case the most highly connected node was the right BA47 (4 connections). In the effective connectivity analysis, we also repeated the finding that this region was significantly predictive of the activity changes taking place in several DMN regions, in particular right BA 31 (beta = 0.19, p = 0.0086), posterior cingulate cortex (beta = 0.28, p = 0.009), left BA 31 (beta = 0.15, p = 0.01), precuneus (beta = 0.39, p = 0.01 corr). Vice-versa, no DMN node had any significant effective connection with right BA47.

#### Group comparison: artists vs non-artists during the Creativity Task

**Figure 3:**
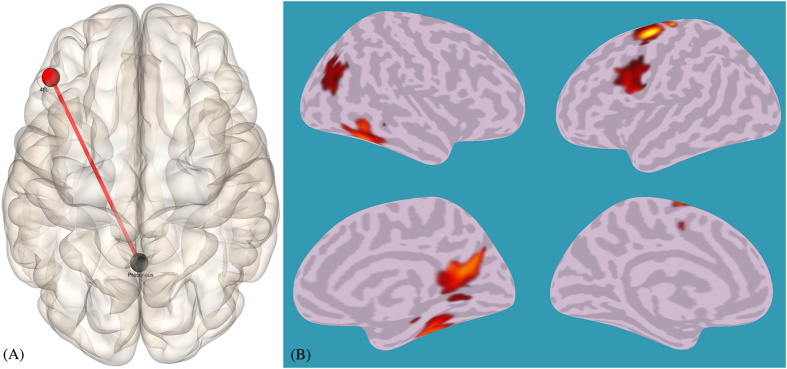
Functional connectivity differences between artists and non-artist in the Creativity Task. (**A**) Results of a ROI-to-ROI analysis using the same regions as in the previous ROI-to-ROI analysis (not differentiating between artists and non-artists), showing a tighter connection between the precuneus (x = 0, y = −56, z = 28) and the left BA46 node (uncorr, t = 3.28, p = 0.005). (**B**) 3D brain surface results from a seed-to-voxel analysis to explore group differences in functional connectivity between the precuneus (x = 0, y = −56, z = 28) and all other voxels of the whole brain. The analysis revealed tighter functional connectivity in artists vs non-artists of the precuneus with right posterior cingulate cortex, left premotor cortex, right parahippocampal cortex/fusiform gyrus, left dorsolateral prefrontal cortex, and right associative visual cortex (see Table 4).

In a last round of analyses, we looked for functional connectivity difference between the two groups (artists and non-artist) in the Creativity Task. First, we performed a ROI-to-ROI analysis (using the same regions as in the previous ROI-to-ROI analysis), which revealed a tighter connection between the Precuneus and left BA46 (uncorr, t = 3.28, p = 0.005; see Fig. 3A). We found no significant difference in the effective connectivity of right BA 47 between artists and non-artists. We proceeded with a seed-to-voxel analysis to explore group differences in functional connectivity between the precuneus (x = 0, y = −56, z = 28), a key node of the DMN that plays a key role in supporting cognitive functions^50^, and all other voxels of the whole brain. The analysis revealed tighter functional connectivity in artists vs non-artists in the precuneus with six different clusters, mainly centered around the following structures: right posterior cingulate cortex, left premotor cortex, right parahippocampal cortex/fusiform gyrus, left dorsolateral prefrontal cortex, right associative visual cortex (see Table 4 and Fig. 3B).

## Discussion

In this study we looked at the functional connectivity of brain activity during a visually creative task. Our main hypothesis was that planning an artwork would influence the functional connectivity between regions part of the DMN, implicated in divergent thinking and generating novel ideas, and of the EN, implicated in evaluating and selecting ideas. We focused on how connectivity within and between these networks differed during a creative task from two control tasks (rest and imagining the alphabet), and we found three main results. First, we indeed found stronger functional connectivity between ENs and DMNs, mainly in the right hemisphere. In particular, we found that the medial frontal node (located in the anterior part of the medial frontal gyrus, Brodmann Area 10), a key DMN node linked to stimulus independent thought (stream of thought unrelated to immediate sensory input)^51^,^52^, was more tightly connected to right EN regions (frontal eye fields and dorsolateral prefrontal) during the creative phase compared to rest. In other studies, this region has been associated with individual differences in creativity^53^,^54^. This finding suggests that creativity can be viewed as a careful balance between top-down executive control and more free-thinking/mind-wandering processes. These findings are consistent with ideas that emphasize the need to balance convergent and divergent thinking during creativity^23^, as well as models of creativity that include phases of idea generation and evaluation^9^. By focusing on connectivity measures, our results argue for the direct interplay between these two networks, rather than successive periods of only executive control followed by only mind wandering. Instead, our pattern of results argues for stronger interactions between these two abilities during the same period of heightened creativity.

Second, we found weaker functional connectivity within the EN towards the right inferior prefrontal gyrus. Given that this region is implicated in inhibitory control^49^, this finding is consistent with the idea of decreased inhibition during creativity. Additionally, with effective connectivity analyses, useful to understand the time-related relationships between connected regions, and how much the activity of a source node is predictive of the activity of a target region, we found a strikingly stronger role of this region in predicting activity in several DMN nodes, rather than the opposite direction. A number of case studies have argued for a role of disinhibition in the tendency of some stroke and degenerative brain disease patients to begin to start to create artwork, often for the first time in decades and often obsessively (for a review, see ref. 55). Recent studies have also shown reduced inhibitory control during musical improvisation compared to mere rote repetition. One example is a recent study that contrasted piano improvisation, such as found in jazz, to playing scales^56^. More generally, studies of musical improvisation, across a number of different styles, have suggested a cooperation between brain networks involved in cognitive control and in free, spontaneous thought^4^,^5^,^39^,^57^.

As a third result, we found differences between professional artists and control participants who did not have extensive training and did not regularly create art. Functional connectivity of the precuneus (a key node of DMN) during the Creative Task was stronger for the artists in connection with other cortical nodes, namely posterior cingulate cortex, premotor cortex, parahippocampal area/fusiform gyrus, dorsolateral prefrontal cortex and associative visual cortex. The link with the left premotor cortex may be explained by the greater expertise of artists in planning artwork not only abstractly, but in physically making art, perhaps through repetitive action schemata.

It is important to note, however, that the artists here differed from the controls in several ways. The artists tested were experts, having spent thousands of hours learning to make visual art and in creating artwork. They had also chosen to become artists, which might also reflect some basic differences in skills or aptitude even before training. Lastly, the artists reported more skill with visual imagery, as measured by the Vividness of Visual Imagery Questionnaire^41^. Based on previous studies, these differences in parahippocampal and fusiform activity might have reflected greater visual imagery of objects, visual features or scenes^58^,^59^,^60^,^61^. Given the current debate regarding talent versus practice in expertise, it could be interesting in future studies to investigate differences between artists and non-artists in a more longitudinal fashion.

These findings suggest that visual creativity may require finding a way for two processes that are often thought of as opposites to work together, namely the free generation of associations and ideas on the one hand (linked to DMN activity), and their evaluation on the other (linked to EN activity). Current literature is coherent with the idea that DMN regions are involved in the generation of novel ideas, thus reflecting stimulus-independent thought that sustain the production of divergent thinking. For example, medial prefrontal cortex has been implicated in insight during problem solving^30^,^62^, or during creative story generation^63^. But this is only one aspect of the processes that define creativity. The other part is the actual selection and evaluation of the ideas generated, and this role might be played by EN, given its role in other aspects of selection and control. EN regions are known to be involved in a variety of creative tasks, such as problem solving^28^,^64^, visual art^65^, divergent thinking^66^, and others. Converging interpretations see the involvement of EN in creativity as supporting goal-directed behavior in order to quickly select and focus on innovative and salient aspects of the generative process^34^,^67^,^68^. Therefore, the concurrent activation of EN and DMN during creative mental effort might be a subtle equilibrium between selection and generation that recruits several brain regions normally working in alternation^53^.

Visual art is widely considered to be one of the pinnacles of human civilization, with its finest examples preserved in museums. Given that art seems to have developed along with the growth in organized societies, it is thought to play an important role in transmitting knowledge and group cohesion. Whether it is visual art, music, or scientific inventions, all creative pursuits involve some mechanisms of problem solving, idea generation, and idea evaluation. These capacities allow us to use art to communicate complex mental abstractions in a concrete and meaningful way that expresses a message to the outside world that is accessible regardless of language, cultural, social, educational, or economic background, and persists across hundreds or thousands of years^69^,^70^. These forms of creativity give us the ability to connect with human culture past and present, and even to peek into the mindset of another individual, by giving us a window into their world regardless of whether their art (in whatever form) was created 10 minutes ago, or 10,000 years ago. What current studies suggest, including the present one, is that in terms of effort and engagement, creativity requires the concurrent contributions of several resources at once, as well as an expert balance of brain networks typically viewed as being in opposition.

## Additional Information

**How to cite this article:** De Pisapia, N. *et al*. Brain networks for visual creativity: a functional connectivity study of planning a visual artwork. *Sci. Rep.* **6**, 39185; doi: 10.1038/srep39185 (2016).

**Publisher's note:** Springer Nature remains neutral with regard to jurisdictional claims in published maps and institutional affiliations.

## Figures and Tables

**Table 1:** MNI coordinates (Montreal Neurological Institute) of the regions of interests in the default mode network (DMN) and in the executive control network (EN).

Region	Acronym	x	y	z	Networks
MEDIAL PREFRONTAL CORTEX	MPFC	−1	49	−5	DMN
POSTERIOR CINGULATE CORTEX	PCC	−6	−52	40	DMN
PRECUNEUS	Precuneus	0	−56	28	DMN
LEFT PRECUNEUS/POSTERIOR CINGULATE CORTEX	31L	−10	−66	24	DMN
RIGHT PRECUNEUS/POSTERIOR CINGULATE CORTEX	31R	10	−66	24	DMN
LEFT LATERAL PARIETAL	LLP	−46	−70	36	DMN
RIGHT LATERAL PARIETAL	RLP	46	−70	36	DMN
LEFT ANTERIOR DORSOLATERAL PREFRONTAL CORTEX	10L	−27	63	6	EN
RIGHT ANTERIOR DORSOLATERAL PREFRONTAL CORTEX	10R	27	63	6	EN
LEFT DORSOLATERAL PREFRONTAL CORTEX	46L	−46	38	12	EN
RIGHT DORSOLATERAL PREFRONTAL CORTEX	46R	46	38	12	EN
LEFT FRONTAL EYE FIELDS	8L	−37	21	44	EN
RIGHT FRONTAL EYE FIELDS	8R	37	21	44	EN
LEFT INFERIOR FRONTAL GYRUS	47L	−40	24	−10	EN
RIGHT INFERIOR FRONTAL GYRUS	47R	40	24	−10	EN

**Table 2:** Functional connectivity (all participants) between EN and DMN nodes in the Creative Task versus Resting State.

	L10	L46	L47	L8	R10	R46	R47	R8	L31	R31	PREC	MPFC	PCC	LLP	RLP
L10		—	—	—	—	—	—	—	—	—	—	—	—	—	—
L46	—		—	—	−3.10; 0.02	—	−4.99; 0.002	—	—	—	—	—	—	—	—
L47	—	—		—	—	—	—	—	—	—	—	—	—	—	—
L8	—	—	—		—	—	—	—	3.23; 0.03	3.02; 0.04	3.02; 0.04	—	—	—	—
R10	—	−3.10; 0.02	—	—		−3.53; 0.02	—	—	5.67; 0.0008	3.96; 0.01	3.35; 0.02	—	5.14; 0.001	—	—
R46	—	—	—	—	−3.53; 0.02		−3.94; 0.02	—	—	—	—	—	—	—	—
R47	—	−4.99; 0.002	—	—	—	−3.94; 0.02		—	5.24; 0.0009	4.67; 0.001	4.40; 0.002	2.51; 0.07	4.00; 0.005	3.33; 0.01	—
R8	—	—	—	—	—	—	—		4.38; 0.003	3.41; 0.01	3.55; 0.01	3.00; 0.02	2.94; 0.02	—	—
L31	—	—	—	3.23; 0.03	5.67; 0.0008	—	5.24; 0.0009	4.38; 0.003		—	—	—	2.58; 0.05	3.35; 0.01	—
R31	—	—	—	3.02; 0.04	3.96; 0.01	—	4.67; 0.001	3.41; 0.01	—		—	—	3.83; 0.006	—	—
PREC	—	—	—	3.02;0.04	3.35; 0.02	—	4.40; 0.002	3.55; 0.01	—	—		—	—	2.92;0.04	—
MPFC	—	—	—	—	—	—	2.51; 0.07	3.00; 0.02	—	—	—		—	—	—
PCC	—	—	—	—	5.14; 0.001	—	4.00; 0.005	2.94; 0.02	2.58; 0.05	3.83; 0.006	2.92; 0.04	—		2.84; 0.04	—
LLP	—	—	—	—	—	—	3.33; 0.01	—	3.35; 0.01	—	—	—	2.84; 0.04		—
RLP	—	—	—	—	—	—	—	—	—	—	—	—	—	—	

(FDR corr, p > 0.05; numbers in each cell are t-value and p-value). For the label of brain regions, L means left, R means right and the number corresponds to the Brodmann area.

**Table 3:** 

	L10	L46	L47	L8	R10	R46	R47	R8	L31	R31	PREC	MPFC	PCC	LLP	RLP
L10		—	—	—	—	—	—	—	—	—	—	—	—	—	—
L46	—		−3.3; 0.01	—	—	—	−6.29; 0.0002	—	—	—	—	—	—	—	—
L47	—	−3.32; 0.01		—	—	—	—	—	—	4.25; 0.01	—	—	—	—	—
L8	—	—	—		—	—	—	—	—	—	—	—	—	—	—
R10	—	—	—	—		—	—	—	—	3.90; 0.02	—	—	—	—	—
R46	—	—	—	—	—		−5.99; 0.0004	—	—	—	—	—	—	—	—
R47	—	−6.29; 0.0002	—	—	—	−5.99; 0.0004		—	3.23; 0.02	—	—	—	3.30; 0.02	—	—
R8	—	—	—	—	—	—	—		—	4.25; 0.01	—	—	—	—	—
L31	—	—	—	—	—	—	3.23; 0.02	—		—	—	—	—	—	—
R31	—	—	—	—	3.90; 0.02	—	4.25; 0.01	4.25; 0.01	—		—	—	—	—	—
PREC	—	—	—	—	—	—	—	—	—	—		—	—	—	—
MPFC	—	—	—	—	—	—	—	—	—	—	—		—	—	—
PCC	—	—	—	—	—	—	3.30; 0.02	—	—	—	—	—		—	—
LLP	—	—	—	—	—	—	—	—	—	—	—	—	—		—
RLP	—	—	—	—	—	—	—	—	—	—	—	—	—	—	

Functional connectivity (all participants) between EN and DMN nodes in the Creative Task versus Alphabet task (FDR corr, p > 0.05; numbers in each cell are t-value and p-value). For the label of brain regions, L means left, R means right and the number corresponds to the Brodmann area.

**Table 4:** 

Cluster	Regions	x	y	z	Total Voxels
1	DORSAL POSTERIOR CINGULATE CORTEX (R)	24	−58	24	440
	CINGULATE CORTEX (R)				
	RETROSPLENIAL CINGULATE CORTEX (R)				
	VENTRAL POSTERIOR CINGULATE CORTEX (R)				
2	PREMOTOR CORTEX (L)	−20	−10	50	334
3	PARAHIPPOCAMPAL CORTEX (R)	36	−36	−26	219
	INFERIOR TEMPORAL GYRUS (R)				
	FUSIFORM GYRUS (R)				
4	FUSIFORM GYRUS (R)	46	−48	−16	190
5	DORSOLATERAL PREFRONTAL CORTEX (L)	−42	2	22	169
	PREMOTOR CORTEX (L)				
6	ANGULAR GYRUS (R)	36	−74	26	159
	ASSOCIATIVE VISUAL CORTEX (R)				

Clusters resulting from a seed-to-voxel analysis to explore Artists vs. Non Artists differences in functional connectivity during the Creative Task (seed in the Precuneus (x = −22, y = −8, z = 58)). The analysis revealed tighter functional connectivity in six different clusters, each covering different brain structures (REGIONS). Coordinates and TOTAL VOXELS refer to the entire cluster.
